# A conceptual clinical reasoning framework for early syndromic recognition in dental practice

**DOI:** 10.3389/froh.2026.1873598

**Published:** 2026-06-26

**Authors:** Abedalrahman Shqaidef, Shantanu Dixit, Maher Al Shayeb, Dinesh Rokaya

**Affiliations:** 1 Clinical Sciences Department, College of Dentistry, Ajman University, Ajman, United Arab Emirates; 2 Center of Medical and Bio-Allied Health Sciences Research, Ajman University, Ajman, United Arab Emirates; 3 Dental Imaging & Diagnostic Center, Ballabgarh, Haryana, India

**Keywords:** clinical reasoning, dental practice, diagnostic framework, genetic syndromes, oral manifestations, sentinel findings, syndromic recognition

## Abstract

**Background:**

Oral and craniofacial findings are reported in more than 900 genetic syndromes, yet their relevance at the chairside is often unclear. Dentists routinely encounter these features as isolated observations, while most available literature—being syndrome-centered—provides limited guidance on when a finding should be considered clinically significant. Delayed recognition of syndromic patterns leads to missed opportunities for early intervention.

**Methods:**

We conducted a narrative review integrating evidence from syndromic dentistry, epidemiology, and clinical reasoning. A sentinel finding was defined as a common oral or craniofacial feature that, when present beyond a defined threshold or in a characteristic pattern, increases the likelihood of an underlying syndromic condition. Using predefined selection criteria, six sentinel findings were identified.

**Results:**

For each finding, we specify when it becomes clinically relevant, outline associated syndromes and genetic links, and indicate what to elicit on history and examination, including extraoral features. These elements are considered together within a four-step analytical pathway, which distinguishes findings suitable for monitoring from those requiring referral.

**Conclusions:**

This feature-based framework is designed to guide decisions when routine findings raise suspicion of an underlying syndrome. By structuring how such features are interpreted, it aims to reduce diagnostic uncertainty and facilitate earlier recognition in practice. The framework remains conceptual and requires prospective validation.

## 1 Introduction

Dentists often encounter oral findings ranging from minor morphological variations to multiple missing teeth, which may represent congenital or acquired, benign or malignant, isolated or syndromic conditions (1, 2). In most cases, these presentations are managed within routine restorative, orthodontic, or periodontal care. However, the same findings, particularly when present in recognizable patterns, may represent early or even sole manifestations of an underlying syndromic condition (3, 4).

A syndrome is defined as a recognizable constellation of symptoms and physical findings that indicate a specific condition, although the underlying cause may not always be known (5). Approximately 900 of more than 5,000 known genetic syndromes involve dental, oral, or craniofacial anomalies (1, 3). Even common findings may warrant reassessment when they deviate from expected patterns. This can create difficulty in distinguishing benign variation from potentially syndromic indicators. The challenge is further compounded by the fact that many syndromic conditions are not clinically evident in early stages, particularly in the absence of a known family history (2).

Clinical reasoning relies on ambiguous clinical cues. Medical education describes two complementary pathways: System 1, which is fast and intuitive, and System 2, which is slower and analytical and is engaged when findings do not align with initial expectations (6, 7). System 1 is susceptible to cognitive biases such as anchoring and confirmation bias, whereas System 2 helps mitigate their influence (8). Structured approaches to diagnostic reasoning remain inconsistently emphasized in dental education, contributing to variability in clinical application (9).

The dental literature has extensively described syndromic conditions and their oral manifestations (10–12). However, most resources remain organized by individual syndromes, offering limited guidance on interpreting specific findings in routine practice. In the absence of defined thresholds, clinicians often rely on memorization of syndrome–feature associations rather than structured evaluation under uncertainty. Evidence is also fragmented across heterogeneous study designs, and existing classification systems are not easily translated to chairside decision-making (1, 11, 13). This reflects a gap between descriptive knowledge and practical clinical reasoning.

To address this gap, we propose a feature-based clinical reasoning framework based on sentinel oral findings. The objectives are to identify oral and craniofacial findings that may serve as sentinel findings; to define evidence-informed thresholds distinguishing benign variation from findings requiring further evaluation; to integrate principles of clinical reasoning and cognitive bias mitigation; and to provide a structured chairside approach for history taking, examination, and referral.

## 2 Diagnostic challenge

**Figure 1:**
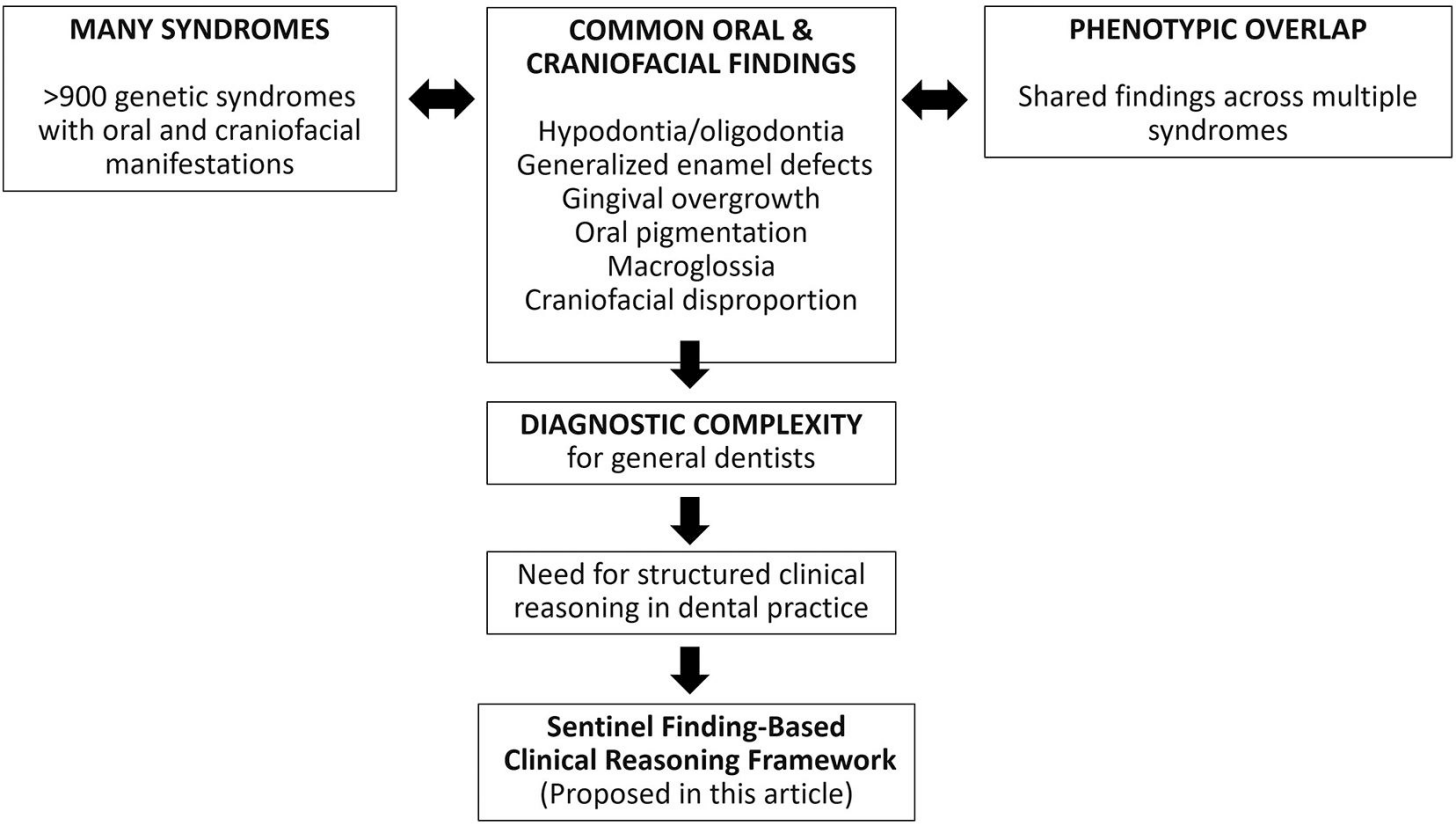
Overlap of common oral and craniofacial manifestations across multiple genetic syndromes.

The relationship between genetic syndromes and oral findings is not one-to-one. As illustrated in Figure 1, numerous syndromes converge on a limited set of shared oral and craniofacial manifestations, creating substantial phenotypic overlap.

Because these findings frequently overlap across multiple syndromic conditions, no single feature is sufficiently specific to reliably indicate a particular syndrome, limiting the practicality of memorization-based approaches in routine dental practice. Figure 1 is an original conceptual illustration developed by the authors to represent the phenotypic overlap of oral and craniofacial findings across syndromic conditions, based on patterns described in the syndromic dentistry literature.

The convergence illustrated in Figure 1 is supported by systematic evidence. Torres-Avalos et al. (10) synthesized oral and craniofacial manifestations across multiple syndromes, demonstrating recurrent overlap across diverse conditions. Consequently, no single finding is pathognomonic; findings such as hypodontia may occur across multiple syndromic conditions, complicating direct syndrome–feature associations in routine dental practice.

The clinical challenge is therefore not the identification of specific syndromes, but recognition of when a finding may indicate a broader syndromic pattern. This underscores the need for structured clinical reasoning to guide suspicion and referral.

Clinical geneticists use “gestalt” pattern recognition, a holistic approach that integrates facial dysmorphisms, clinical features, and patient history to recognize syndromic patterns and generate diagnostic hypotheses (14, 15). Artificial intelligence tools such as Face2Gene and GestaltMatcher further support this process by analyzing facial phenotypes to prioritize candidate syndromes (16, 17). However, their application in routine dental practice remains exploratory, with limitations including potential algorithmic bias, limited validation in dental populations, and lack of integration into everyday clinical workflows. Accordingly, such tools should be considered adjunctive rather than diagnostic. Ethical considerations, including data privacy, informed consent, algorithmic bias, cybersecurity concerns, and unresolved legal and implementation challenges, further constrain the current applicability of AI-assisted facial phenotyping and underscore that such tools should be considered adjunctive rather than replacements for specialist clinical assessment (18).

When a single finding corresponds to multiple conditions, clinicians require a structured approach to support decision-making under uncertainty. The proposed framework adapts principles of structured pattern recognition to dental practice through sentinel oral findings. These concepts form the basis of the framework, and the following section outlines its methodological development.

## 3 Methods: framework development

### 3.1 Review design

This is a narrative review with conceptual framework development. It integrates evidence from epidemiological, syndromic, and cognitive literature to build a clinical reasoning model, rather than aiming for an exhaustive synthesis of a narrowly defined question. The intent is to bring these domains into a single, clinically usable framework.

### 3.2 Literature search and selection strategy

A purposive literature search was conducted to support framework development rather than to comprehensively retrieve all available studies. Searches were performed in PubMed/MEDLINE and Scopus, supplemented by targeted searches in Google Scholar to identify seminal and highly cited works. The search focused on three complementary domains:

1. **Evidence related to syndromic recognition in dental practice:** Feature-based search terms (e.g., hypodontia, enamel hypoplasia, gingival overgrowth) combined with “syndrome”, “genetic disorder”, and “oral manifestations”.
2. **Epidemiological baseline data:** Population-based studies reporting prevalence and presentation patterns of selected findings in the general population.
3. **Clinical reasoning theory:** Foundational literature on diagnostic reasoning, dual-process theory, and cognitive bias in clinical decision-making.

**Search details (narrative approach):** The search was not restricted by a specific start date; however, priority was given to literature published from 2010 onwards to capture contemporary evidence. Initial searches were completed in January 2025 and supplemented iteratively during framework development.

General search term combinations used in PubMed/MEDLINE and Scopus included: for syndromic findings, (hypodontia OR oligodontia OR enamel hypoplasia OR gingival overgrowth OR oral pigmentation OR macroglossia OR craniofacial abnormalities) AND (syndrome OR genetic disorder OR oral manifestations); for epidemiological evidence, (prevalence OR incidence) combined with the selected finding terms; and for clinical reasoning, (diagnostic reasoning OR dual process theory OR cognitive bias) AND (dentistry OR medicine).

Literature was selected based on relevance to framework objectives, methodological robustness, and clinical applicability, with priority given to systematic reviews, meta-analyses, large cohort studies, and clinically relevant reviews. Case reports were not prioritised unless they described rare syndromic associations insufficiently represented in higher-level evidence. Consistent with the narrative review design, no formal quality appraisal tools were applied; instead, evidence was appraised pragmatically according to methodological appropriateness, consistency with the broader literature, and relevance to framework development. Selection and interpretation of evidence occurred iteratively through author discussion and consensus during framework development.

### 3.3 Identification and selection of sentinel findings

A *sentinel finding* is defined as a common oral or craniofacial feature that is typically benign in isolation but, when present at a defined level of severity or in a characteristic pattern, increases the likelihood of an underlying syndromic condition. Definitions of severity level and characteristic pattern for each sentinel finding were informed by literature-derived thresholds where available (e.g., ≥3 missing teeth for hypodontia) and by author consensus in areas where quantitative evidence was limited (e.g., generalized enamel defects affecting multiple teeth). Operationally, such findings function as triggers for structured clinical reasoning when predefined thresholds or patterns are identified.

**Selection Process:** Selection followed a structured, multi-stage approach:

1. **Preliminary scoping:** A broad list of oral and craniofacial findings associated with syndromic conditions was generated through literature review and preliminary scoping of commonly reported syndromic oral and craniofacial findings.
2. **Iterative filtering:** Each finding was evaluated for clinical relevance, detectability, and strength of syndromic association.
3. **Author consensus refinement:** Findings were refined through iterative author consensus to improve consistency, clinical applicability, and alignment with predefined selection criteria. The final sentinel findings were selected only after unanimous agreement among the authors.

**Selection Criteria:** To function as effective triggers for clinical reasoning, each candidate finding was evaluated against the following five criteria. A finding was included if it met at least three:

1. **Early detectability:** Identifiable during routine dental examination, including clinical inspection and standard radiography.
2. **Signal-to-noise ratio:** Clear distinction between normal variation and clinically significant presentation.
3. **Syndromic association:** Documented association with multiple syndromic conditions in the literature.
4. **Actionability:** Recognition leads to a defined clinical action (e.g., targeted history, further evaluation, referral).
5. **Gateway function:** Triggers evaluation of associated extraoral or systemic features.

The criteria were developed through synthesis of the syndromic, epidemiological, and clinical reasoning literature and refined through author consensus to ensure applicability in routine dental practice. The five criteria were selected to balance clinical observability, syndromic relevance, and practical usability in routine dental settings. Requiring fulfillment of at least three criteria was intended to balance sensitivity (capturing clinically meaningful findings) with specificity (excluding highly prevalent or low-actionability findings), while maintaining framework usability.

Selection decisions were cross-checked across epidemiological, syndromic, and clinical reasoning literature to ensure consistency across domains. Disagreements were resolved through iterative consensus with explicit justification based on predefined criteria. Although no formal weighted scoring system was applied, candidate findings were systematically evaluated against the predefined selection criteria (early detectability, signal-to-noise ratio, syndromic association, actionability, gateway function). Final inclusion was determined through iterative author consensus, with emphasis on maintaining both clinical usability and syndromic relevance. This process was intended to balance reproducibility with pragmatic framework development.

A preliminary list of candidate sentinel findings was compiled from the broader literature on oral and craniofacial manifestations of syndromic conditions, including recent evidence syntheses such as Torres-Avalos et al. (10). The structured evaluation matrix for candidate sentinel findings, including rationales for inclusion, supportive status, or exclusion, is provided in Supplementary Table S1.

### 3.4 Final set and scope of sentinel findings

Application of the above criteria resulted in the selection of six sentinel findings:

1. Hypodontia/oligodontia
2. Generalized enamel defects
3. Unexplained gingival overgrowth
4. Oral mucosal pigmentation
5. Macroglossia
6. Craniofacial disproportion

These findings were selected to provide coverage across major oral and craniofacial structures while maintaining a focused framework intended to be clinically usable, subject to prospective validation.

The selection of these six sentinel findings was informed by their relative frequency in general dental practice, documented associations with syndromic conditions in the literature, clinical observability during routine examination, and practical actionability (i.e., the ability to define thresholds for suspicion and referral pathways). The selected findings are not exhaustive; other clinically relevant features (e.g., supernumerary teeth, specific craniofacial dysmorphisms, skeletal asymmetries) may also indicate syndromic conditions but were excluded to maintain framework focus and clinical usability.

### 3.5 Scope clarification: excluded findings

Application of the selection criteria led to exclusion of certain findings commonly described in syndromic literature. This was necessary to maintain focus on ambiguous findings that require clinical reasoning. Excluded categories included:

- **Overt major anomalies (e.g., cleft palate):** Typically mandate immediate referral and do not require diagnostic ambiguity resolution.
- **Highly prevalent, low-specificity conditions (e.g., isolated malocclusion, bruxism):** Limited utility due to low signal-to-noise ratio.
- **Findings not reliably assessable at chairside:** Require specialized diagnostic methods beyond routine practice.
- **Conditions with established specialty pathways:** Already governed by clear clinical guidelines.

Supernumerary teeth were not included as primary sentinel findings. Although associated with certain syndromes, they are often incidental, frequently require radiographic identification, and show inconsistent diagnostic specificity across syndromic conditions. Accordingly, they are considered supportive rather than primary triggers within this framework. In this framework, “primary triggers” are findings that independently warrant structured clinical reassessment when predefined thresholds are met, whereas “supportive triggers” contribute to pattern completion but do not alone initiate the clinical reasoning pathway.

### 3.6 Data synthesis and framework construction

 Data from the three literature domains were integrated for each sentinel finding: (i) epidemiological data informed baseline prevalence and typical presentation, (ii) syndromic evidence-informed threshold selection and associated clinical patterns, and (iii) clinical reasoning theory informed the structure of the diagnostic pathway, emphasizing structured analytical reassessment when clinical findings raise concern.

Data integration was conducted through narrative synthesis, in which findings from the epidemiological, syndromic, and clinical reasoning literature were collated for each sentinel finding and interpreted through iterative author consensus to identify clinically relevant thresholds, associated patterns, and decision points. No formal quantitative or meta-analytic methods were applied. Where quantitative evidence was unavailable or inconsistent, thresholds were defined using literature-informed clinical reasoning and author consensus, and should therefore be regarded as provisional and hypothesis-generating.

This integration resulted in a four-step clinical pathway: **Observation → Threshold Assessment → Pattern Completion → Clinical Action.** Each sentinel finding is linked to a structured clinical decision point and a suggested referral pathway when further evaluation is warranted.

To improve transparency in framework development, the sources informing the framework were differentiated into three categories. First, literature-derived evidence informed prevalence estimates, syndromic associations, and threshold selection where published evidence was available. Second, expert consensus among the authors informed operational definitions and thresholds in areas where quantitative evidence was limited or absent, particularly for findings lacking standardized diagnostic cut-offs. Third, original conceptual contributions by the authors included the five selection criteria for sentinel findings and the proposed four-step clinical reasoning pathway, which were developed through synthesis of the clinical reasoning literature and adapted for dental practice. Operational definitions for each sentinel finding, including diagnostic criteria and thresholds, are provided in the clinical guide (Table 1).

**Table 1:** Sentinel findings clinical guide.

Sentinel findings	Baseline/prevalence	Threshold for suspicion	Associated extra-oral clues	Relevant history clues	Representative syndromes	Early diagnostic evidence	Clinical action/referral	References
Hypodontia/Oligodontia	Hypodontia ≈6.4%; Oligodontia 0.08%–0.36%	≥3 missing teeth raises suspicion; ≥6 missing teeth strongly predictive of syndromic association; Missing teeth in typically stable tooth positions (maxillary central incisors, mandibular canines, first molars) suggests syndromic involvement	**HED—**sparse hair, nail dystrophy, conical teeth, maxillary retrusion; **EVCS**—polydactyly, short stature, labiogingival adhesions, accessory frenula, enamel hypoplasia; **ARS**—iris hypoplasia; **Witkop—**nail dysplasia, conical teeth; **OFCD**—facial dysmorphism	Family history of missing teeth; heat intolerance; developmental delay; parental consanguinity	HED, ARS, Witkop, EVCS, OFCD, BCDS; Down, Apert, Crouzon, Goldenhar	Mean number of missing permanent teeth (syndrome-specific): HED (22.4), ARS (14.3), Witkop (16.75), EVCS (8.5), OFCD (9), BCDS (15.4)	Referral to clinical genetics or a pediatric specialist; genetic testing, if indicated, to be arranged by the specialist	(10, 19, 21, 22)
Generalized Enamel Defects	Global prevalence of developmental defects of enamel (DDE) in primary dentition ranges from 15%–49%; molar-incisor hypomineralization affects 13.1% globally; generalized enamel defects reported in multiple syndromic conditions	Generalized enamel defects affecting multiple teeth; involvement of both dentitions; pattern inconsistent with common environmental causes (fluorosis, MIH); presence of extra-oral findings	**TDOS—**curly/coarse hair; **Junctional epidermolysis bullosa**—skin blistering, nail dystrophy; **Hypohidrotic ectodermal dysplasia—**sparse hair, nail dystrophy, conical teeth; **Focal dermal hypoplasia (Goltz syndrome)—**skin atrophy, syndactyly, nail changes; **Cleidocranial dysplasia—**palpable clavicular defects, hypertelorism; **Ellis–van Creveld—**polydactyly, short stature, nail hypoplasia	Family history of enamel defects or syndromes; consanguinity; history of skin fragility/blistering; delayed tooth eruption; spontaneous dental abscesses	TDOS; Enamel-renal syndrome; Jalili syndrome; Junctional epidermolysis bullosa; Hypohidrotic ectodermal dysplasia; Focal dermal hypoplasia; Ellis–van Creveld syndrome	Generalized enamel defects occur across several hereditary syndromes; amelogenesis imperfecta may present as isolated or syndromic; enamel phenotype varies according to underlying genetic mutation	Referral to clinical genetics or pediatric specialist; multidisciplinary dental care required	(10, 23–25)
Unexplained Gingival Overgrowth	Gingival enlargement not associated with plaque or medications. Incidence varies by cause; genetic forms (isolated and syndromic) are very rare, with prevalence of one case per 750,000 people	Progressive firm fibrotic gingival enlargement; typically generalized; persists despite adequate oral hygiene (bleeding on probing <10%); not medication-induced (phenytoin, cyclosporine, nifedipine); onset commonly during tooth eruption (mixed or permanent dentition)	**Zimmermann–Laband**—coarse facial features, nail hypoplasia/aplasia, joint hyperextensibility; **Ramon**—cherubism, hypertrichosis, short stature; **Cowden—**macrocephaly, facial papules (trichilemmomas), acral keratoses; **Cross**—hypertrichosis, intellectual disability; **Enamel-renal syndrome**—gingival enlargement with dental abnormalities	Family history of gingival enlargement; childhood or adolescent onset; medication history to exclude drug-induced enlargement; history of seizures or developmental delay; personal or family history of breast/thyroid cancers (Cowden); history of renal disease or kidney stones	Zimmermann-Laband syndrome; Ramon syndrome; Cowden syndrome; Enamel-renal syndrome; Cross syndrome; Costello syndrome	Systematic review of 146 pediatric HGF cases reported extra-oral manifestations in 61% of patients and a syndromic association in ∼34%; enamel-renal syndrome consistently reported with gingival fibromatosis and ectopic gingival calcifications	Referral to clinical genetics for syndromic evaluation; targeted genetic testing arranged by specialist; multidisciplinary care depending on suspected syndrome; gingivectomy may be required for functional or aesthetic concerns with possible recurrence	(10, 26–32)
Oral Mucosal Pigmentation	Global pooled prevalence 20.8% (meta-analysis, 70,668 individuals)	Multiple lesions (73% of syndromic cases); lip (38%) or buccal mucosa (31%) location; brown macules (69%); early onset (often childhood); not explained by physiological, amalgam, smoking, or drug-related causes; syndromic pigmentation rare (149 cases across 9 syndromes)	**LHS—**nail pigmentation (≈60% cases), acral sites, no systemic findings; **PJS—**perioral and digital macules (no nail involvement); **MAS—**café-au-lait spots with irregular “coast of Maine” borders; **Carney complex—**lentigines, blue nevi, facial myxomas; **Sturge-Weber—**unilateral facial port-wine stain	Age of onset (childhood vs. adult); family history of pigmentation or GI/breast cancer; abdominal pain or GI bleeding (PJS); fatigue, weight loss, salt craving (Addison); history of adrenalectomy (Nelson syndrome); seizures or developmental delay (Sturge-Weber)	Peutz-Jeghers syndrome; Laugier-Hunziker syndrome; McCune-Albright syndrome; Carney complex; Addison disease; LEOPARD syndrome/Noonan syndrome with multiple lentigines; Sturge-Weber syndrome	Systematic review of 149 cases reported oral pigmentation preceding syndrome diagnosis in 74.5% of cases; PJS pigmentation appears in childhood years before gastrointestinal symptoms; LHS diagnosis of exclusion with no malignant potential	Referral to clinical genetics; gastroenterology for PJS cancer surveillance; endocrinology for Addison disease or MAS; dermatology evaluation for suspicious solitary lesions; bleeding precautions during dental procedures in Sturge-Weber syndrome	(10, 33–36)
Macroglossia	True macroglossia is rare in the general population; one pediatric study reported macroglossia in 24% of children presenting with tongue disorders	Marked tongue protrusion beyond the alveolar ridge at rest or during speech; enlarged or elongated tongue with lateral crenations; diastema or posterior tooth spacing; mandibular protrusion/Class III tendency; tongue interposition between teeth. Exclude pseudomacroglossia (edentulism, tonsillar hypertrophy, low palatal vault) and non-syndromic causes such as vascular malformations or tumors.	**Beckwith-Wiedemann**—nevus flammeus (forehead/eyelids), linear earlobe indentations, maxillary hypoplasia, hemihypertrophy; **Down syndrome—**fissured tongue, midface hypoplasia, upslanting palpebral fissures; **MEN 2B**—multinodular tongue, mucosal neuromas (lips, eyelids), medullated corneal nerves; **Neurofibromatosis type I**—café-au-lait spots, neurofibromas, ± tongue enlargement due to plexiform neurofibroma; **Hurler syndrome**—coarse facial features, corneal clouding, joint stiffness; **Hunter syndrome—**coarse facial features, behavioral differences, no corneal clouding	Age of onset (infancy/childhood vs. adult); feeding difficulties in infancy; noisy breathing or snoring (OSA); speech difficulties; family history of similar features; history of neonatal hypoglycemia (Beckwith-Wiedemann); history of thyroid disease or acromegaly (endocrine workup); edentulous state	Beckwith-Wiedemann syndrome; Down syndrome; MEN 2B; Neurofibromatosis type 1; Hurler syndrome; Hunter syndrome	Macroglossia is a characteristic feature of Beckwith-Wiedemann and Down syndromes; in Beckwith-Wiedemann syndrome it may present early with neonatal hypoglycemia and increased tumor risk; diagnosis is primarily clinical and supported by characteristic morphological patterns (multinodular in MEN 2B, pebbly in lymphangioma, unilateral in hemifacial hyperplasia), which help guide differential diagnosis	Referral to clinical genetics for syndromic evaluation; pediatric referral for Beckwith-Wiedemann (tumor surveillance, hypoglycemia management); ENT/sleep medicine referral for obstructive sleep apnea; endocrinology referral if endocrine disorder suspected; orthodontic evaluation for malocclusion; surgical reduction (partial glossectomy) may be required in severe cases	(10, 31, 37)
Craniofacial disproportion								
Craniosynostosis	Affects 5.9 per 10,000 live births overall; nonsyndromic craniosynostosis accounts for 5.2 per 10,000. Nonsyndromic cases are more common and typically single-sutured, whereas syndromic cases are often multi-sutured.	Premature suture fusion with abnormal head shape (scaphocephaly, trigonocephaly, brachycephaly); palpable ridging along sutures	**Apert—**syndactyly, midface hypoplasia, proptosis; **Crouzon—**proptosis, beaked nose, hypertelorism; **Pfeiffer—**broad thumbs/toes, possible syndactyly; **Saethre-Chotzen—**facial asymmetry, ptosis, low hairline, brachydactyly; **Muenke—**hearing loss, thimble-like middle phalanges	Family history; developmental delay; hearing loss; vision problems (proptosis, strabismus); airway concerns (OSA)	Apert; Crouzon; Pfeiffer; Muenke; Saethre-Chotzen	Syndromic forms account for 15%–30% of all craniosynostosis cases; the five listed syndromes comprise >90% of syndromic cases	Referral to multidisciplinary craniofacial team (neurosurgery, plastic surgery, ENT, genetics, orthodontics)	(10, 38–41)
Maxillary/Midface Hypoplasia	Generally occurs as part of a syndrome; population prevalence of isolated forms not well established	Midface retrusion with concave facial profile; class III malocclusion tendency; flat malar region; depressed nasal bridge; relative mandibular prognathism	**Apert/Crouzon—**proptosis, hypertelorism, parrot-beaked nose; **Down—**upslanting palpebral fissures, epicanthic folds, depressed nasal bridge; **XLHED—**frontal bossing, saddle nose, periorbital hyperpigmentation, prominent lips	Family history; airway concerns (snoring, OSA); hearing loss; vision problems; recurrent otitis media	Apert; Crouzon; Down; achondroplasia; XLHED	Most commonly syndromic; isolated forms rare; midface involvement is characteristic of multiple craniofacial syndromes	Referral to craniofacial team; orthodontic evaluation; ENT evaluation for airway/hearing; genetics consultation	(10, 42)
Mandibular Hypoplasia/Micrognathia	Congenital micrognathia occurs in ∼1.5% of NICU admissions; among hospitalized infants with micrognathia, 7.9% have a genetic syndrome diagnosed neonatally	Micrognathia/retrognathia visible at rest; feeding difficulties in infancy; airway concerns (stridor, OSA); U-shaped cleft palate; glossoptosis	**Treacher Collins—**malformed ears, zygomatic hypoplasia, downslanting palpebral fissures, coloboma; **Goldenhar—**facial asymmetry, ear anomalies, epibulbar dermoids; **Stickler—**cleft palate, hearing loss, high myopia; **Nager**—thumb anomalies, radial ray defects; **Pierre Robin—**micrognathia, glossoptosis, cleft palate (triad)	Family history; feeding difficulties; airway problems; hearing loss; vision problems; maternal polyhydramnios	Pierre Robin sequence; Treacher Collins; Stickler; Nager; Goldenhar (OAV spectrum)	∼93% syndromic (only 6.8% isolated in a surgical series) (44); >60 syndromes associated; among hospitalized infants, 7.9% had a genetic syndrome diagnosed neonatally, though likely an underestimate	Urgent ENT/pediatric referral for airway/feeding assessment; multidisciplinary craniofacial team; genetics consultation; audiology/ophthalmology as indicated	(10, 43–46)

HED, hypohidrotic ectodermal dysplasia; ARS, Axenfeld–Rieger syndrome; EVCS, ellis–van creveld syndrome, OFCD, Oculofaciocardiodental syndrome; BCDS, branchio-oculofacial syndrome; TDOS, tricho-dento-osseous syndrome; EB, epidermolysis bullosa; MIH, molar-incisor hypomineralization; NPIGO, non-plaque-induced gingival overgrowth; HGF, hereditary gingival fibromatosis; LHS, laugier-hunziker syndrome; PJS, peutz-jeghers syndrome; MAS, mccune-albright syndrome; GI, gastrointestinal; BWS, beckwith-wiedemann syndrome; MEN 2B, multiple endocrine neoplasia type 2B; NF1, neurofibromatosis type 1; OSA, obstructive sleep apnea; XLHED—X-linked hypohidrotic ectodermal dysplasia; OAV, oculo-auriculo-vertebral spectrum; CFM, Craniofacial microsomia; ENT, ear, nose, and throat.

### 3.7 Scope and intent

This framework is intended as a cognitive aid to support clinical suspicion and referral, not as a diagnostic tool. Syndromes referenced are illustrative rather than exhaustive. The objective is to support early recognition and facilitate appropriate interdisciplinary referral; definitive diagnosis remains the responsibility of relevant medical specialties.

### 3.8 Operationalization of the clinical reasoning framework

The framework presented here defines sentinel findings as clinical triggers for structured reassessment of initial impressions. Each finding is linked to evidence-informed thresholds intended to promote structured clinical reassessment and reduce anchoring bias. A “pattern completion” step is included to guide evaluation of associated and discordant features, reducing the risk of confirmation bias and premature closure. The aim is not syndrome identification, but recognition of when clinical suspicion is justified. This approach supports focused history taking, targeted examination, and appropriate interdisciplinary referral when indicated.

## 4 Clinical reasoning framework

### 4.1 Overview of the clinical reasoning pathway

Clinical decision-making in dentistry operates through complementary cognitive modes: intuitive (System 1) and analytical (System 2) reasoning (6). This dual-process model offers a useful conceptual analogy for understanding why syndromic findings may be overlooked in routine practice—for instance, when rapid, intuitive reasoning (System 1) may contribute to premature classification of oral anomalies as normal variation—and how a more analytical approach (System 2) may support structured reassessment (6, 8).

**Figure 2:**
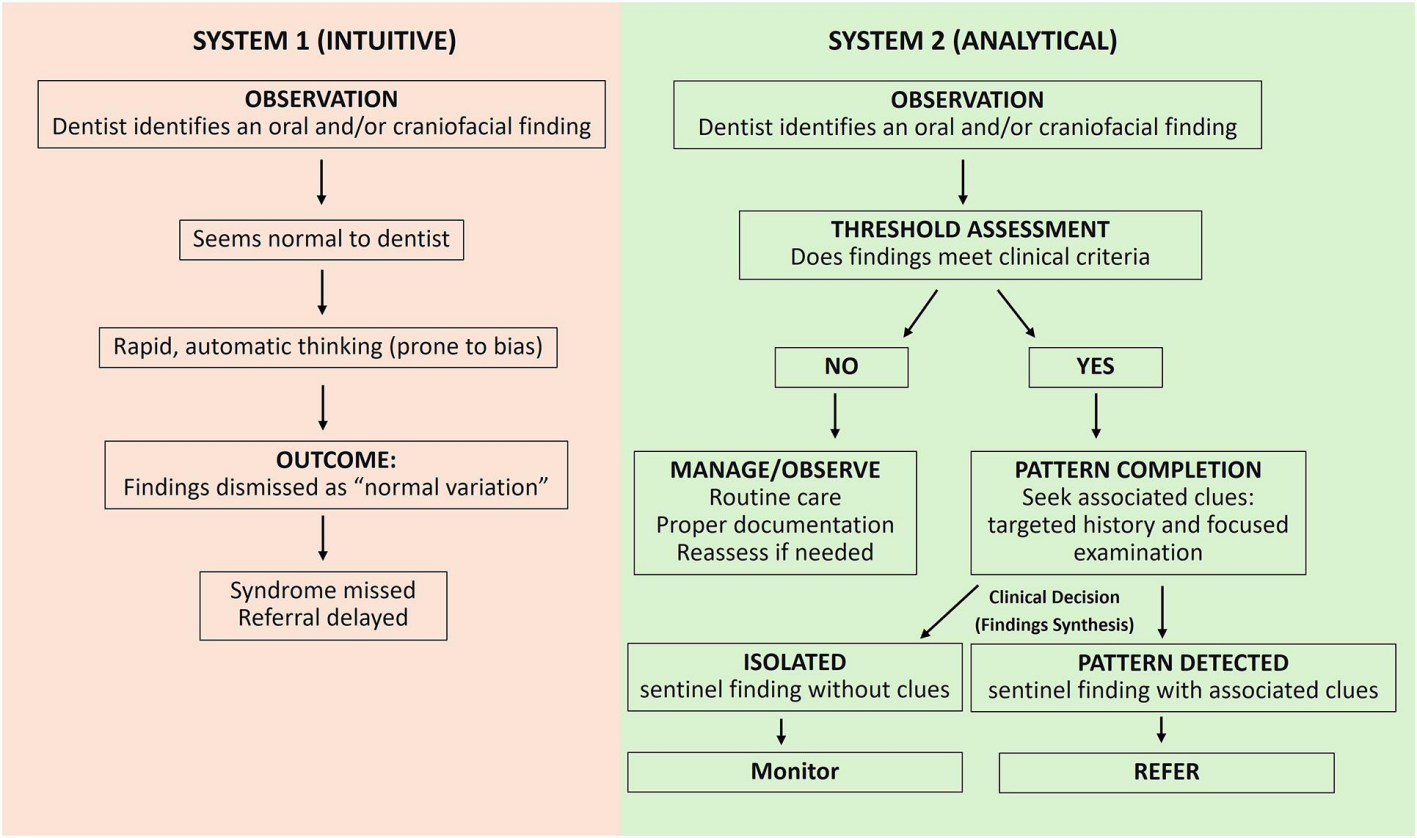
Comparison of intuitive (system 1) and analytical (system 2) clinical reasoning pathways in syndromic recognition.

Figure 2 illustrates the distinction between these two pathways. As a conceptual model, System 1 reasoning represents rapid, experience-based thinking that may contribute to premature classification of findings that fall outside expected physiological or developmental variation as normal variation. In contrast, System 2 reasoning represents more deliberate, analytical thinking that may support more careful interpretation of uncertain findings and help mitigate cognitive bias. In Figure 2, the System 1 pathway (left) reflects rapid, experience-based pattern recognition, whereas the System 2 pathway (right) represents the proposed four-step analytical framework (Observation → Threshold Assessment → Pattern Completion → Clinical Action), intended to support more deliberate evaluation and timely referral when syndromic patterns are suspected. The framework is conceptually derived from dual-process theory (6).

The analytical pathway consists of four sequential steps intended for chairside use:

1. **Observation**: An oral or craniofacial finding is identified during routine examination.
2. **Threshold Assessment:** The finding is evaluated to determine whether its severity, distribution, or pattern exceeds what is typically expected in general practice, using evidence-informed thresholds for each sentinel finding, derived from published literature where available and supplemented by author consensus (**see the clinical guide in** Table 1).
  - If the finding falls within expected variation, it is managed as an isolated developmental or acquired feature with routine documentation and follow-up.
  - If the finding exceeds expected variation, it is considered a sentinel finding and the evaluation proceeds to the next step.
3. **Pattern Completion**: A focused search is performed to determine whether the finding is isolated or part of a broader feature pattern. This involves targeted history-taking covering systemic, developmental, and family history, along with a brief extraoral and soft tissue examination for associated findings. The aim is not to establish a diagnosis but to determine whether further evaluation is warranted.

**Clinical Action**: Management is guided by the synthesis of findings:

- If the sentinel finding is isolated, it is documented and managed with routine care and periodic monitoring.
- If associated features are identified, referral to an appropriate specialist (e.g., paediatrician, clinical geneticist, or dermatologist) is advised. The referral should be clearly documented, and patients or caregivers should be informed that it is a precautionary step for further evaluation.

This structured pathway reframes syndromic recognition from an intuitive process into a structured clinical workflow. By following these four steps, clinicians move from initial observation to justified clinical action in a consistent and reproducible manner.

### 4.2 Application to sentinel findings

The following sections operationalize the four-step reasoning pathway for each of the six sentinel findings summarized in the clinical guide (Table 1). For each finding, epidemiological baselines, evidence-informed thresholds, targeted history, focused examination, and associated extraoral features are integrated into a structured clinical guide. Each section culminates in a clearly defined clinical action—distinguishing between findings that can be monitored and those that warrant referral for further evaluation.

In the structured guide that follows, each sentinel finding is presented with a standardized clinical definition, epidemiological context, evidence-informed thresholds for suspicion (high-risk findings/red flags), targeted history and examination, associated extraoral clues (supportive findings), and a clearly defined clinical action. Findings exceeding defined thresholds function as primary referral triggers, whereas associated clues serve as supportive findings that contribute to pattern completion and clinical suspicion. To improve practical readability, a simplified chairside summary checklist derived from the framework is provided in Supplementary Table S2.

#### 4.2.1 Hypodontia/oligodontia

(a) **Definition and clinical presentation**: Hypodontia is the developmental absence of one or more permanent teeth (excluding third molars). Oligodontia is the absence of six or more teeth, and anodontia the complete absence of teeth; the term hypodontia is often used generically for any degree of tooth agenesis (19, 20). Tooth agenesis can occur as an isolated trait (non-syndromic) or as part of a syndrome (21).
(b) **Baseline prevalence**: In the general population, the prevalence of hypodontia (excluding third molars) is approximately 6.4% (95% CI: 5.7–7.2), with wide geographic variation. The highest prevalence is reported in Africa (13.4%), followed by Europe (7.0%), Asia and Australia (6.3% each), and North America (5.0%) (19). Oligodontia in the permanent dentition is much rarer, with a prevalence of 0.08%–0.36% (19, 20).
(c) **Threshold for suspicion**: A finding of **three or more** congenitally missing permanent teeth (excluding third molars) should raise suspicion for a possible underlying syndrome. Six or more missing teeth is strongly predictive of a syndromic association (19, 21). In addition, absence of teeth in typically stable positions (maxillary central incisors, mandibular canines, first molars) is rare in non-syndromic hypodontia and often indicates a syndromic cause (21).
(d) **Representative syndromic associations (archetypes)**: Numerous syndromes feature hypodontia or oligodontia as a prominent sign. The following represent archetypal examples rather than an exhaustive list.
  - **Hypohidrotic ectodermal dysplasia (HED)**—X-linked or autosomal dominant; hallmark triad of hypodontia/oligodontia, sparse hair, and reduced sweating; conical anterior teeth are a diagnostic clue (12, 21).
  - **Axenfeld–Rieger syndrome (ARS)**—autosomal dominant; ocular dysgenesis, maxillary hypoplasia, and dental anomalies including oligodontia, microdontia, taurodontism (21, 47).
  - **Witkop tooth and nail syndrome**—autosomal dominant; hypodontia/oligodontia associated with nail dysplasia (21, 48).
  - **Ellis–van Creveld syndrome (EVCS)**—autosomal recessive; skeletal dysplasia, polydactyly, and oligodontia with conical teeth, labiogingival adhesions (21).
  - **Branchio-oculofacial syndrome (BCDS)**—autosomal dominant; facial clefting, euryblepharon, and oligodontia (21).
  - **Oculofaciocardiodental syndrome (OFCD)**—X-linked dominant; congenital cataracts, facial dysmorphism, and oligodontia with radiculomegaly (21).
  - Other syndromes often associated include Down syndrome, Crouzon, Apert, and Goldenhar (2, 10).
(e) **Targeted history**: A thorough history should explore:
  - **Family history** of missing teeth, tooth shape anomalies, or diagnosed syndromes (autosomal dominant patterns are common) (20).
  - **Developmental history:** delayed tooth eruption, feeding difficulties, speech issues.
  - **Systemic symptoms:** heat intolerance or reduced sweating (HED), ocular abnormalities (ARS), nail dystrophy (Witkop), polydactyly or short stature (EVCS), skin fragility (junctional epidermolysis bullosa) (12).
  - **Parental consanguinity** (relevant for autosomal recessive conditions) (21).
(f) **Focused examination:**
  - **Integumentary:** inspect scalp hair, eyebrows, eyelashes for sparseness; assess nails for dystrophy; note any hypohidrosis or dry skin.
  - **Craniofacial:** evaluate for frontal bossing, maxillary retrusion, depressed nasal bridge, periorbital hyperpigmentation, and ear abnormalities.
  - **Intraoral:** record pattern of missing teeth (using panoramic radiograph), note conical or peg-shaped teeth, microdontia, enamel defects, and any labiogingival frenula or cleft palate (12, 21).
(g) **Associated clues (pattern completion)**: When hypodontia meets the threshold, the presence of any of the following extraoral clues strengthens the likelihood of an underlying syndrome:
  - **HED:** sparse hair, absent eyebrows, heat intolerance, conical teeth.
  - **ARS:** iris hypoplasia, corectopia, maxillary hypoplasia.
  - **Witkop:** nail dystrophy, conical teeth.
  - **EVCS:** polydactyly, short stature, accessory labiogingival frenula, enamel hypoplasia.
  - **BCDS:** facial clefting, ectropion, euryblepharon.
  - **OFCD:** congenital cataracts, radiculomegaly, facial dysmorphism.
(h) **Clinical action and referral:**
  - **Isolated hypodontia** (<3 teeth, no extraoral findings): document findings, provide routine restorative/orthodontic care, and monitor at recall visits.
  - **Sentinel pattern** (≥3 missing teeth, or presence of extraoral clues): refer to clinical genetics or a pediatrician for comprehensive evaluation. Genetic testing to be arranged by the specialist (12, 20, 21).
  - **Oligodontia** (≥6 missing teeth) should prompt referral regardless of other findings, as syndromic associations are highly prevalent (19).

#### 4.2.2 Generalized enamel defects

(a) **Definition and clinical presentation**: For the purpose of this framework, generalized enamel defects refer to developmental enamel abnormalities affecting multiple teeth across different quadrants and extending beyond the localized patterns typically expected from common environmental or acquired causes (e.g., fluorosis, molar-incisor hypomineralisation, localized trauma, tetracycline staining). These defects may include quantitative abnormalities (hypoplasia) or qualitative abnormalities (hypomineralization and hypomaturation), often involving both dentitions. Such presentations can occur as isolated traits (e.g., amelogenesis imperfecta) or as part of a syndromic condition (12, 23).
(b) **Baseline prevalence**: Globally, developmental defects of enamel (DDE) in the primary dentition affect 15%–49% of children (24), and molar-incisor hypomineralization (MIH) has a global prevalence of 13.1% (25). Syndromic enamel defects are much rarer, but their recognition is critical because the oral findings may be the first clue to an underlying genetic disorder (10).
(c) **Threshold for suspicion:** Generalized enamel defects affecting multiple teeth in both dentitions should raise suspicion for a syndromic cause, particularly when the distribution, severity, or morphology of the defects is inconsistent with common environmental or acquired causes (e.g., fluorosis typically affects homologous teeth symmetrically; molar-incisor hypomineralisation (MIH) is generally limited to first permanent molars and incisors; tetracycline staining is diffuse and uniform but not associated with enamel hypoplasia). The presence of additional dental anomalies (e.g., hypodontia, taurodontism, delayed eruption) or extraoral findings (e.g., hair or nail abnormalities, skin fragility) further strengthens syndromic suspicion (12, 23).
(d) **Representative syndromic associations (archetypes):**
  - **Tricho-dento-osseous syndrome (TDOS)**—autosomal dominant; curly/coarse hair, bone sclerosis, and thin enamel with taurodontism (23).
  - **Junctional epidermolysis bullosa (JEB)**—autosomal recessive; skin blistering, nail dystrophy, and generalized enamel hypoplasia (12).
  - **Hypohidrotic ectodermal dysplasia (HED)**—sparse hair, nail dystrophy, conical teeth, and enamel defects (12).
  - **Focal dermal hypoplasia (Goltz syndrome)**—X-linked dominant; skin atrophy, syndactyly, nail changes, and enamel hypoplasia (12).
  - **Cleidocranial dysplasia (CCD)**—autosomal dominant; clavicular hypoplasia, delayed suture closure, and enamel hypoplasia (23).
  - **Ellis–van Creveld syndrome (EVCS)**—autosomal recessive; polydactyly, short stature, and enamel hypoplasia (10).
(e) **Targeted history:**
  - **Family history** of enamel defects, early tooth loss, or known syndromes.
  - **Consanguinity** (relevant for autosomal recessive conditions).
  - **History of skin fragility/blistering** (suggests epidermolysis bullosa).
  - **Delayed tooth eruption** or spontaneous dental abscesses.
  - **Bone pain, fractures, or short stature** (consider associated skeletal involvement).
(f) **Focused examination:**
  - **Extraoral:** assess hair texture and density, nail quality, skin integrity (blisters, scars, hyperkeratosis), facial dysmorphism (e.g., frontal bossing, saddle nose, micrognathia).
  - **Intraoral:** evaluate the pattern, distribution, and severity of enamel defects using clinical and radiographic examination. Note the presence of hypodontia, taurodontism, or pulp calcifications. Panoramic radiography helps compare radiodensity of enamel and dentine.
(g) **Associated clues (pattern completion):**
  - **TDOS:** curly hair, bone sclerosis, taurodontism.
  - **JEB:** skin blisters, nail dystrophy, scarring.
  - **HED:** sparse hair, heat intolerance, conical teeth.
  - **Goltz syndrome:** skin atrophy, syndactyly, nail dystrophy.
  - **CCD:** palpable clavicular defects, delayed fontanelle closure, supernumerary teeth.
  - **EVCS:** polydactyly, short stature, labiogingival adhesions.
(h) **Clinical action and referral**
  - Isolated enamel defects (consistent with AI, no extraoral findings): refer to a pediatric dentist and/or orthodontist for restorative planning and lifelong monitoring.
  - Sentinel pattern (generalized defects with extraoral findings): refer to clinical genetics for syndromic evaluation. Genetic testing may be indicated.
  - Multidisciplinary care involving pediatric dentistry, orthodontics, and restorative dentistry is essential for functional and aesthetic rehabilitation (23).

#### 4.2.3 Unexplained gingival overgrowth

(a) **Definition and clinical presentation**: In this framework, “unexplained gingival overgrowth” is used as a clinical working category to identify patients with progressive, generalized gingival enlargement that persists after exclusion of common local (plaque-induced inflammation) and drug-induced causes (e.g., phenytoin, cyclosporine, nifedipine). The term is not intended as a formal diagnostic entity but rather as a practical trigger for syndromic consideration. Hereditary gingival fibromatosis (HGF) is the most common syndromic presentation and typically manifests as generalized, firm, pink, non-hemorrhagic gingival enlargement that may partially or completely cover the teeth (27, 28).
(b) **Baseline prevalence**: Genetic forms (isolated and syndromic) are very rare, with a prevalence of approximately one case per 750,000 people (30). Among pediatric patients with HGF, 33.8% present with syndromic HGF (26).
(c) **Threshold for suspicion**: Progressive firm, fibrotic gingival enlargement that is generalized, persists despite adequate plaque control and gingival health (e.g., bleeding on probing <10%), and is not associated with medications known to induce overgrowth (e.g., phenytoin, cyclosporine, nifedipine). Onset commonly coincides with eruption of the permanent dentition (26, 27, 32).
(d) **Representative syndromic associations (archetypes):**
  - **Zimmermann-Laband syndrome**—coarse facial features, nail hypoplasia/aplasia, joint hyperextensibility (29).
  - **Ramon syndrome**—cherubism, hypertrichosis, short stature (29).
  - **Cowden syndrome**—macrocephaly, facial papules (trichilemmomas), acral keratoses, increased risk of breast and thyroid cancers (30).
  - **Enamel-renal syndrome**—gingival fibromatosis, amelogenesis imperfecta, nephrocalcinosis (29).
  - **Neurofibromatosis type 1**—café-au-lait spots, neurofibromas; gingival enlargement may occur (28).
(e) **Targeted history:**
  - **Family history** of gingival enlargement or syndromes.
  - **Age of onset** (childhood or adolescence favors genetic causes).
  - **Medication history** (to exclude drug-induced overgrowth).
  - **History of seizures** (may suggest phenytoin use or underlying neurological disorder).
  - **Personal or family history** of breast, thyroid, or renal cancers (Cowden syndrome).
  - **History of renal disease** or kidney stones (enamel-renal syndrome).
(f) **Focused examination:**
  - **Extraoral:** assess for coarse facies, hypertrichosis, macrocephaly, skin lesions (café-au-lait spots, neurofibromas, papules), nail abnormalities, joint hyperextensibility.
  - **Intraoral:** grade the extent of gingival overgrowth using standard indices; note whether it is generalized or localized, and evaluate for associated dental anomalies (e.g., enamel defects, delayed eruption, malocclusion). Panoramic radiographs may reveal ectopic calcifications, impacted teeth, or bone changes.
(g) **Associated clues (pattern completion):**
  - **Zimmermann-Laband:** nail hypoplasia, coarse facies, joint laxity.
  - **Ramon:** cherubism, hypertrichosis, short stature.
  - **Cowden:** trichilemmomas, macrocephaly, cancer history.
  - **Enamel-renal:** enamel defects, renal calcifications.
  - **NF1:** café-au-lait spots, neurofibromas.
(h) **Clinical action and referral:**
  - **Isolated HGF** (no extraoral findings): refer to periodontist for management; surgical gingivectomy/gingivoplasty may be required. Long-term follow-up is essential because recurrence rates are high (48% at 3 years) (26).
  - **Syndromic suspicion** (presence of extraoral features): refer to **clinical genetics** for evaluation. Targeted genetic testing may be arranged.
  - **Multidisciplinary care:** depending on the syndrome, involvement of dermatology, endocrinology, or oncology may be needed.

#### 4.2.4 Oral mucosal pigmentation

(a) **Definition and clinical presentation**: Oral mucosal pigmentation can be broadly classified as physiological (ethnic pigmentation), acquired (exogenous: e.g., amalgam tattoo, smoker's melanosis, drug-induced; endogenous: e.g., endocrine disorders, post-inflammatory pigmentation), or syndromic, where pigmentation occurs in association with multisystem disorders (e.g., Peutz-Jeghers syndrome, McCune-Albright syndrome) (36). While physiologic pigmentation is common, syndromic pigmentation typically presents as multiple, well-circumscribed brown or black macules that may be isolated to the mouth or associated with cutaneous and systemic findings (33).
(b) **Baseline prevalence**: The global pooled prevalence of oral pigmented lesions is 20.8% (35), but syndromic pigmentation is rare; a systematic review identified 149 cases across nine syndromes (33).
(c) **Threshold for suspicion**: Suspicion should be raised when pigmentation is:
  - **Multiple** (73% of syndromic cases).
  - Located on **lips** (38%) or **buccal mucosa** (31%).
  - **Brown** in color (69%) (33).
  - Onset in **childhood** or early adulthood.
  - Not explained by physiological (ethnic) pigmentation, amalgam tattoo, smoking, or drugs.
  - Accompanied by extraoral pigmentation or systemic symptoms.
(d) **Representative syndromic associations (archetypes):**
  - **Peutz-Jeghers syndrome (PJS)**—autosomal dominant; perioral and digital brown macules, intestinal hamartomatous polyps, increased cancer risk (31).
  - **Laugier-Hunziker syndrome (LHS)**—acquired; mucosal and nail pigmentation, no systemic findings, no malignant potential (10).
  - **McCune-Albright syndrome (MAS)**—polyostotic fibrous dysplasia, café-au-lait spots with irregular (“coast of Maine”) borders, endocrine abnormalities.
  - **Carney complex**—lentigines, blue nevi, cardiac myxomas, endocrine tumors.
  - **LEOPARD syndrome/Noonan syndrome with multiple lentigines**—multiple lentigines, cardiac defects, hypertelorism.
  - **Sturge-Weber syndrome**—unilateral facial port-wine stain, ocular and neurological involvement; oral pigmentation may occur (31, 49).
(e) **Targeted history:**
  - **Age of onset** (childhood suggests PJS; adult onset may indicate LHS).
  - **Family history** of pigmentation, polyps, or cancers (PJS).
  - **Gastrointestinal symptoms** (abdominal pain, bleeding, intussusception)—suggestive of PJS.
  - **Systemic symptoms:** seizures, developmental delay (Sturge-Weber).
  - **History of adrenalectomy** (Nelson syndrome).
(f) **Focused examination:**
  - **Extraoral:** examine skin for pigmented macules (perioral, digital, palms/soles), café-au-lait spots, port-wine stain, lentigines. Inspect nails for longitudinal melanonychia (LHS).
  - **Intraoral:** document location, number, size, and color of pigmented macules. Lesions are typically flat and non-blanching. Biopsy is indicated if any lesion is suspicious for melanoma.
(g) **Associated clues (pattern completion)**
  - **PJS:** perioral and digital pigmentation, intestinal polyps, family history.
  - **LHS:** nail pigmentation, no systemic findings, no family history.
  - **MAS:** polyostotic fibrous dysplasia, precocious puberty, irregular café-au-lait spots.
  - **Carney complex:** cardiac myxomas, lentigines, endocrine neoplasia.
  - **Addison:** hyperpigmentation in palmar creases, scars, mucosal surfaces, hypotension.
(h) **Clinical action and referral**
  - **LHS:** reassure patient; no malignancy risk; treat for cosmetic concerns if desired.
  - **PJS:** refer to **clinical genetics** and **gastroenterology** for cancer surveillance (colonoscopy, upper endoscopy). Screen for breast, ovarian, and testicular tumors (33).
  - **MAS:** refer to **endocrinology** and **clinical genetics**; monitor for endocrine dysfunction.
  - **Addison:** urgent endocrinology referral.
  - **Sturge-Weber:** neurology and ophthalmology involvement; bleeding precautions during dental procedures.
  - **Any solitary or changing pigmented lesion:** dermatology referral for biopsy to rule out melanoma.

#### 4.2.5 Macroglossia

(a) **Definition and clinical presentation:** Macroglossia is an abnormally large tongue that may be true (tissue overgrowth) or relative (apparent enlargement due to a small oral cavity). Clinical consequences include feeding difficulties, speech impairment, airway obstruction, and dental deformities such as anterior open bite, diastemas, and mandibular prognathism (10, 12, 31, 50).
(b) **Baseline prevalence**: True macroglossia is rare; in one pediatric study of children with tongue disorders, macroglossia was reported in 24%. It is most often syndromic (37).
(c) **Threshold for suspicion**
  - Marked tongue protrusion beyond the alveolar ridge at rest or during speech.
  - Enlarged or elongated tongue with lateral crenations.
  - Diastema or posterior tooth spacing.
  - Mandibular protrusion or Class III tendency.
  - Tongue interposition between teeth.
  - Before considering macroglossia as a sentinel finding, alternative explanations should be systematically excluded through focused clinical assessment. These include pseudomacroglossia (relative tongue enlargement due to reduced oral cavity volume, e.g., edentulism, tonsillar hypertrophy, low palatal vault) and localised non-syndromic causes (e.g., vascular malformations, tumours). Exclusion should be based on history and clinical examination, including inspection, palpation, and assessment of oral anatomy. If a non-syndromic explanation is identified, the finding should not be considered a primary trigger for syndromic evaluation (31, 37).
(d) **Representative syndromic associations (archetypes)**
  - **Beckwith-Wiedemann syndrome (BWS)**—neonatal hypoglycemia, omphalocele, hemihypertrophy, linear earlobe indentations, nevus flammeus (12, 31).
  - **Down syndrome (DS)**—midface hypoplasia, upslanting palpebral fissures, intellectual disability (10, 31).
  - **Multiple endocrine neoplasia type 2B (MEN 2B)**—multinodular tongue, mucosal neuromas (lips, eyelids), medullated corneal nerves, medullary thyroid carcinoma (31, 49).
  - **Neurofibromatosis type 1 (NF1)**—café-au-lait spots, neurofibromas; tongue enlargement may occur from plexiform neurofibroma (31, 51).
  - **Mucopolysaccharidoses (Hurler, Hunter)**—coarse facies, corneal clouding, joint stiffness, enlarged tongue (12, 51).
(e) **Targeted history:**
  - **Age of onset** (infancy/childhood suggests BWS, DS, or mucopolysaccharidoses; adult onset may indicate MEN 2B).
  - **Feeding difficulties** in infancy.
  - **Noisy breathing or snoring** (suggests obstructive sleep apnea).
  - **Speech difficulties**.
  - **Family history** of similar features.
  - **Neonatal hypoglycemia** (BWS).
(f) **Focused examination:**
  - **Extraoral:** evaluate for dysmorphic features (midface hypoplasia, macrocephaly, hemihypertrophy, coarse facies). Inspect skin for café-au-lait spots, neurofibromas, and perioral neuromas.
  - **Intraoral:** measure tongue size relative to oral cavity; note lateral crenations, mucosal neuromas (MEN 2B), pebbly appearance (lymphangioma). Assess dental arch form, spacing, and occlusion. Evaluate airway (stridor, noisy breathing).
(g) **Associated clues (pattern completion):**
  - **BWS:** neonatal hypoglycemia, earlobe creases, hemihypertrophy, omphalocele.
  - **Down syndrome:** flat facial profile, upslanting palpebral fissures, single palmar crease.
  - **MEN 2B:** mucosal neuromas, medullated corneal nerves, marfanoid habitus.
  - **NF1:** café-au-lait spots, axillary freckling, neurofibromas.
  - **Hurler/Hunter:** coarse facies, corneal clouding, joint contractures, hepatosplenomegaly.
(h) **Clinical action and referral**
  - **Syndromic suspicion:** refer to **clinical genetics** for evaluation.
  - **Pediatric referral:** for BWS (tumor surveillance, hypoglycemia management).
  - **ENT/sleep medicine:** for obstructive sleep apnea evaluation.
  - **Orthodontic evaluation:** for malocclusion management.
  - **Surgical reduction** (partial glossectomy) may be required in severe cases with airway obstruction or functional impairment.

#### 4.2.6 Craniofacial disproportion

In this framework, “craniofacial disproportion” refers to clinically significant deviations from expected craniofacial proportions that exceed normal developmental or familial variation and are associated with syndromic conditions. Mild variations in facial proportions that fall within expected developmental or familial limits are not considered sentinel findings. The following sub-sections describe specific disproportionate craniofacial patterns (craniosynostosis, maxillary/midface hypoplasia, mandibular hypoplasia) that, when exceeding defined thresholds or occurring with associated extraoral features, warrant syndromic consideration.

##### 4.2.6.1 Craniosynostosis

(a) **Definition and clinical presentation**: Craniosynostosis is the premature fusion of one or more cranial sutures, leading to an abnormal skull shape and, in syndromic forms, associated facial and systemic anomalies. Syndromic craniosynostosis accounts for 15%–30% of all cases and is often multisutural (39, 52).
(b) **Baseline prevalence**: Craniosynostosis affects approximately 5.9 per 10,000 live births overall. Non-syndromic forms (single-suture) are more common; syndromic forms are often multi-sutural (39).
(c) **Threshold for suspicion**: Premature suture fusion with abnormal head shape (scaphocephaly, trigonocephaly, brachycephaly) and palpable ridging along sutures. Presence of additional craniofacial or systemic anomalies suggests a syndromic cause (39, 41).
(d) **Representative syndromic associations (archetypes):**
  - **Apert syndrome**—bicoronal craniosynostosis, syndactyly, midface hypoplasia, proptosis (11, 52).
  - **Crouzon syndrome**—bicoronal craniosynostosis, proptosis, beaked nose, hypertelorism (41, 52).
  - **Pfeiffer syndrome**—bicoronal craniosynostosis, broad thumbs/toes, midface hypoplasia (41).
  - **Muenke syndrome**—coronal synostosis, macrocephaly, hearing loss (10, 41).
  - **Saethre-Chotzen syndrome**—coronal synostosis, facial asymmetry, ptosis, low hairline, brachydactyly (39, 41, 51).
(e) **Targeted history:**
  - **Family history** of craniosynostosis or related syndromes.
  - **Developmental delay**.
  - **Hearing loss**.
  - **Vision problems** (proptosis, strabismus).
  - **Airway concerns** (snoring, OSA).
(f) **Focused examination:**
  - **Cranial:** head circumference, shape, palpation of sutures, fontanelle status.
  - **Facial:** midface hypoplasia, proptosis, hypertelorism, ear position, cleft palate.
  - **Extremities:** syndactyly, broad thumbs/toes, brachydactyly.
  - **Neurological:** developmental milestones, suspected raised intracranial pressure (e.g., papilledema) warrants medical referral.
(g) **Associated clues (pattern completion):**
  - **Apert:** syndactyly, midface hypoplasia.
  - **Crouzon:** proptosis, beaked nose, normal hands.
  - **Pfeiffer:** broad thumbs/toes.
  - **Muenke:** macrocephaly, hearing loss.
  - **Saethre-Chotzen:** low hairline, ptosis, small ears.
(h) **Clinical action and referral:**
  - **Suspected syndromic craniosynostosis:** refer to a **multidisciplinary craniofacial team** (neurosurgery, plastic surgery, ENT, genetics, orthodontics) for evaluation and management. Early surgical intervention may be required to prevent increased intracranial pressure and optimize neurodevelopment (39).

##### 4.2.6.2 Maxillary/midface hypoplasia

(a) **Definition and clinical presentation**: Maxillary hypoplasia refers to a primary skeletal deficiency of the maxilla, resulting in retrusion of the middle third of the face and often associated with dentoalveolar and/or mandibular compensatory changes (e.g., Class III malocclusion). In contrast, midface hypoplasia is a broader term describing deficiency of the midfacial skeleton, which may involve the maxilla, zygomatic complex, and nasal-orbital structures.

In this framework, maxillary/midface hypoplasia is considered a sentinel finding when the degree of disproportionality exceeds expected developmental variation or is accompanied by associated syndromic features. Representative underlying conditions can be broadly grouped into: craniosynostosis syndromes (e.g., Apert, Crouzon, Pfeiffer), chromosomal disorders (e.g., Down syndrome), skeletal dysplasias (e.g., achondroplasia), and ectodermal dysplasias (e.g., X-linked hypohidrotic ectodermal dysplasia).

b. **Baseline prevalence**: Generally occurs as part of a syndrome; population prevalence of isolated forms is not well established.
c. **Threshold for suspicion**: Further syndromic investigation should be considered when maxillary or midface hypoplasia presents as disproportionate midfacial retrusion relative to mandibular position (e.g., concave facial profile, tendency toward Class III malocclusion) that exceeds expected developmental or familial variation (42), particularly when accompanied by one or more of the following: (i) unexplained skeletal Class III pattern or cephalometric evidence of maxillary deficiency; (ii) associated functional features such as chronic nasal obstruction, persistent oral breathing, or sleep-disordered breathing; or (iii) additional syndromic features (e.g., craniosynostosis, ocular proptosis, hypertelorism, ear abnormalities, digital anomalies) or relevant family history. Isolated mild retrusion without functional impairment or syndromic features is not considered a sentinel finding within this framework.
d. **Representative syndromic associations (archetypes):**
  - **Apert/Crouzon**—proptosis, hypertelorism, parrot-beaked nose.
  - **Down syndrome**—upslanting palpebral fissures, epicanthic folds, depressed nasal bridge.
  - **X-linked hypohidrotic ectodermal dysplasia (XLHED)**—frontal bossing, saddle nose, periorbital hyperpigmentation, prominent lips (31, 42, 49, 51).
e. **Targeted history:**
  - **Family history** of similar facial features or syndromes.
  - **Airway concerns** (snoring, OSA) due to midface narrowing.
  - **Hearing loss** (common in craniosynostosis syndromes).
  - **Vision problems** (proptosis, strabismus).
  - **Recurrent otitis media**.
f. **Focused examination:**
  - **Facial profile:** assess concavity, nasal bridge, malar projection.
  - **Intraoral:** evaluate occlusion (class III tendency, crossbite), high-arched palate.
  - **Cephalometric analysis** may be required for orthodontic and surgical planning.
g. **Associated clues (pattern completion):**
  - **Apert/Crouzon:** craniosynostosis, proptosis, syndactyly (Apert).
  - **Down:** epicanthal folds, intellectual disability, single palmar crease.
  - **Achondroplasia:** rhizomelic dwarfism, trident hand.
  - **XLHED:** sparse hair, hypodontia, heat intolerance.
h. **Clinical action and referral:**
  - **Referral to a craniofacial team or clinical genetics** for diagnosis.
  - **Orthodontic evaluation** for management of class III malocclusion.
  - **ENT evaluation** for airway obstruction, hearing loss.
  - **Surgical management** (e.g., Le Fort osteotomy) may be indicated in adulthood after growth completion (42).

##### 4.2.6.3 Mandibular hypoplasia/micrognathia

(a) **Definition and clinical presentation**: Mandibular hypoplasia refers to true skeletal underdevelopment of the mandible, which may involve reduced ramus and/or body length, altered gonial angle, and overall deficient mandibular growth. In contrast, micrognathia is a descriptive morphological term referring to a small mandible, which may result from true skeletal hypoplasia, positional factors (e.g., intrauterine constraint), or syndromic conditions. In this framework, mandibular hypoplasia and micrognathia are considered together as potential sentinel findings; however, true skeletal hypoplasia carries greater syndromic suspicion, particularly when progressive or accompanied by associated anomalies. Mandibular hypoplasia may occur in isolation or as part of a sequence (e.g., Pierre Robin sequence) with glossoptosis and airway compromise. It frequently occurs in syndromic conditions and may contribute to feeding difficulties and respiratory compromise in infancy.
(b) **Baseline prevalence**: Congenital micrognathia is a common reason for NICU admission (1.5% of infants in a multicenter cohort). Among hospitalized infants with micrognathia, 7.9% had a genetic syndrome diagnosed neonatally (43). In a surgical series, approximately 93% of patients with congenital mandibular hypoplasia had an associated syndrome, with only 6.8% being nonsyndromic (44).
(c) **Threshold for suspicion:** A finding of mandibular hypoplasia or micrognathia should prompt further syndromic investigation when one or more of the following are present:
  - **Progressive morphological deviation**—worsening mandibular retrusion, asymmetry, or failure to demonstrate expected mandibular growth over time.
  - **Functional consequences**—airway compromise (e.g., stridor, obstructive sleep apnea, cyanotic episodes), feeding difficulties (poor suck, choking, prolonged feeding, nasogastric or gastrostomy tube requirement), or occlusal instability (e.g., severe overjet, anterior open bite).
  - **Associated anomalies**—cleft palate (particularly U-shaped), glossoptosis, ear abnormalities, ocular anomalies, cardiac defects, or limb abnormalities.
  - **Syndromic features or family history**—associated craniofacial stigmata (e.g., malar hypoplasia, downslanting palpebral fissures, thumb anomalies) or a family history of craniofacial syndromes.

Isolated, non-progressive micrognathia without functional impairment or associated anomalies (e.g., positional retrognathia) is not considered a sentinel finding within this framework.

d. **Representative syndromic associations (archetypes):**
  - **Pierre Robin sequence**—micrognathia, glossoptosis, U-shaped cleft palate; may be isolated or syndromic (e.g., Stickler, Treacher Collins) (31, 43).
  - **Treacher Collins syndrome**—mandibular hypoplasia, malformed ears, zygomatic hypoplasia, downslanting palpebral fissures, coloboma (43, 49).
  - **Stickler syndrome**—cleft palate, hearing loss, high myopia, joint hypermobility (43).
  - **Goldenhar syndrome (OAV spectrum)**—facial asymmetry, ear anomalies, epibulbar dermoids (53).
  - **Nager syndrome**—mandibular hypoplasia, thumb anomalies, radial ray defects (53).
  - **22q11.2 deletion syndrome**—micrognathia, cleft palate, cardiac defects, immune deficiency (43).
e. **Targeted history**
  - **Family history** of craniofacial anomalies or syndromes.
  - **Feeding difficulties** in infancy (duration, need for tube feeding).
  - **Airway problems** (stridor, apnoeic episodes, snoring).
  - **Hearing loss**.
  - **Vision problems** (myopia, retinal detachment in Stickler).
  - **Maternal polyhydramnios** (due to impaired fetal swallowing).
f. **Focused examination:**
  - **Extraoral:** profile assessment (micrognathia, retrognathia), ear morphology, facial asymmetry, zygomatic prominence, palpebral fissures, coloboma.
  - **Intraoral:** evaluate palate for cleft (U-shaped or V-shaped), tongue position (glossoptosis), and airway patency.
  - **Airway assessment:** auscultation, pulse oximetry, sleep study if indicated.
g. **Associated clues (pattern completion):**
  - **Pierre Robin:** isolated or part of Stickler, Treacher Collins, 22q11.2 deletion.
  - **Treacher Collins:** malformed ears, coloboma, zygomatic hypoplasia.
  - **Stickler:** high myopia, vitreoretinal degeneration, joint hypermobility.
  - **Goldenhar:** epibulbar dermoids, preauricular tags, vertebral anomalies.
  - **Nager:** thumb/radial anomalies.
h. **Clinical action and referral:**
  - **Newborns** with micrognathia and respiratory distress: urgent ENT/pediatric referral for airway management (positioning, nasopharyngeal airway, possible mandibular distraction or tracheostomy).
  - **Feeding support:** gastroenterology referral for gastrostomy tube if oral feeding is inadequate (32.7% of hospitalized infants required gastrostomy) (43).
  - **Multidisciplinary craniofacial team:** for definitive management (orthodontics, orthognathic surgery, speech therapy).
  - **Genetics consultation:** to identify underlying syndrome.
  - **Audiology and ophthalmology** evaluations as indicated (e.g., Stickler, Treacher Collins).

## 5 Discussion

This narrative review presents a feature-based clinical reasoning framework designed to help general dentists recognize when common oral and craniofacial findings may signal an underlying syndromic condition. The framework is based on the observation that multiple syndromic conditions may converge on a limited set of shared oral and craniofacial findings, creating substantial phenotypic overlap that may complicate chairside recognition.

Table 2 summarizes representative syndromic conditions associated with shared oral and craniofacial findings, illustrating the substantial phenotypic overlap that characterizes syndromic recognition.

**Table 2:** Representative oral and craniofacial findings with associated syndromic conditions [adapted from Torres-Avalos et al. (10)].

Craniofacial and oral findings	Representative syndromes
Craniosynostosis	Crouzon, Apert, Pfeiffer, Muenke, Saethre-Chotzen, Jackson-Weiss
Midface/mandibular hypoplasia	Pierre Robin, Treacher Collins, Hallermann-Streiff, Goldenhar, Cornelia de Lange, Nager, Down, Turner, Edwards, Rubinstein-Taybi, Robinow, DiGeorge, Muenke
Dental anomalies (hypodontia, oligodontia, enamel hypoplasia)	Down, Crouzon, Apert, Treacher Collins, Ellis-van Creveld, Papillon-Lefèvre, Rieger, Ectodermal Dysplasia, Cleidocranial Dysostosis, Marfan, Gardner, Cornelia de Lange, Goldenhar, Rubinstein-Taybi, Sotos, Robinow
Gingival hypertrophy/fibromatosis	Zimmermann-Laband, Ramon, H syndrome, Cowden syndrome
Mucocutaneous pigmentation	Peutz-Jeghers, Laugier-Hunziker
Macroglossia	Beckwith-Wiedemann, Down, Angelman, Pierre Robin, Robinow, Williams

This convergence highlights an important diagnostic challenge: isolated findings rarely correspond to a single syndromic entity and may instead represent overlapping phenotypic expressions across multiple conditions.

Table 3 summarizes the key clinical implications of phenotypic overlap and highlights the rationale for a finding-based clinical reasoning approach in general dental practice.

**Table 3:** Clinical implications of phenotypic overlap.

Observation	Implications
**Convergence**: Numerous syndromes share a limited set of findings	A single finding cannot reliably predict a specific syndrome
**Overlap**: The same finding appears across multiple conditions	One-to-one mapping is not feasible in practice
**Complexity**: Multiple findings intersect across syndromes	Pattern recognition must guide clinical suspicion

Together, these observations provide the conceptual basis for a structured framework centered on six sentinel oral findings: hypodontia/oligodontia, generalized enamel defects, unexplained gingival overgrowth, oral mucosal pigmentation, macroglossia, and craniofacial disproportion. Each finding is linked to evidence-informed thresholds and associated clinical clues. The framework provides a structured clinical pathway intended to support systematic clinical suspicion and referral decision-making in general dental practice. Rather than enabling definitive syndromic diagnosis, the model is intended to reduce diagnostic uncertainty and facilitate timely referral. Conceptual frameworks such as this represent an essential step prior to prospective validation in clinical settings and are widely recognized as a necessary precursor to the development and validation of clinical decision-support tools. The framework is hypothesis-generating and requires prospective validation to assess its diagnostic accuracy, impact on referral patterns, and clinical utility. A key limitation is that the framework has not yet been empirically validated, and its performance characteristics—including diagnostic accuracy, reproducibility across clinicians, and real-world utility—remain unknown. If validated in future prospective studies, this framework could contribute to earlier syndromic recognition and reduced diagnostic delays; however, such population-level benefits remain hypothetical and require empirical confirmation.

### 5.1 Comparison with existing literature

Numerous studies have catalogued the oral and craniofacial manifestations of genetic syndromes. Comprehensive reviews report that over 900 syndromes involve dental or craniofacial anomalies (1, 10), and detailed descriptions of individual conditions are widely available in databases and textbooks (2, 13, 31, 46, 49). While these resources are valuable, they are primarily syndrome-centric or descriptive compilations of features. They outline what may occur but provide limited guidance on when a commonly encountered finding should raise suspicion, how to systematically evaluate associated features, or what clinical action should follow.

The present framework addresses this translational gap by shifting the focus from syndrome memorization to structured clinical reasoning. It operationalizes principles from cognitive psychology and medical education—dual-process theory (6) and illness scripts (54)—into a practical chairside tool. By defining explicit, evidence-informed thresholds (e.g., three or more missing teeth in hypodontia) and embedding them within a stepwise analytical pathway (Observation → Threshold Assessment → Pattern Completion → Clinical Action), the framework is intended to encourage more deliberate analytical reasoning when findings exceed expected variation, drawing on dual-process theory as a conceptual guide.

Understanding clinical reasoning as a dynamic interplay between intuitive and analytical processes (7) further supports this approach. Moreover, diagnostic categories—including syndromes—are conceptual tools whose value lies in their ability to organize clinical experience and guide decision-making (5). The present framework aligns with this perspective by offering a clinically actionable model rather than attempting to redefine syndromic classification.

### 5.2 Implications for dental practice and education

The framework is designed for practical application in routine dental care. The six sentinel findings are commonly encountered, yet their potential syndromic significance is often overlooked. The structured pathways and associated cues may function as chairside cognitive aids or be integrated into electronic health records as decision-support prompts. Earlier recognition of syndromic patterns enables timely multidisciplinary management, potentially improving patient outcomes and reducing delays in diagnosis (1, 2). For example, a child presenting with generalized enamel defects affecting both dentitions together with delayed eruption and hair abnormalities may exceed the proposed threshold for syndromic suspicion, prompting targeted history-taking and consideration of multidisciplinary referral. Such examples illustrate how the framework is intended to support structured clinical suspicion rather than diagnostic determination.

In dental education, this framework offers a structured method to teach diagnostic reasoning and cognitive bias awareness. Evidence suggests that formal training in diagnostic reasoning remains underdeveloped in dental curricula (9). Introducing threshold-based evaluation and pattern completion can help students develop more robust illness scripts, reducing reliance on intuitive shortcuts that may contribute to diagnostic error (8). This aligns with the broader view that clinical judgment in dentistry requires integration of both intuitive and analytical reasoning processes (55).

Importantly, the framework also functions as a cognitive safeguard. Explicit thresholds help counteract anchoring bias, where clinicians may prematurely interpret findings as normal variation. The emphasis on pattern completion encourages the active search for disconfirming evidence, reducing confirmation bias and promoting more balanced clinical judgment (6).

The clinical relevance of this approach is supported by empirical data. A recent cross-sectional study of 213 adolescents with genetically confirmed syndromes reported that 68% exhibited dental anomalies, with hypodontia as the most common finding. Notably, nearly half of the patients demonstrated multiple co-occurring anomalies, reinforcing the importance of pattern-based assessment rather than reliance on isolated findings (3).

The framework is intended to support clinical suspicion and structured referral decision-making, rather than provide definitive syndromic diagnosis.

## 6 Limitations

Several limitations should be acknowledged. First, this work represents a narrative review with conceptual framework development rather than a systematic review. Although a structured and purposive search strategy was employed, the thresholds and associations proposed are based on the best available evidence and may require refinement as new data emerge. Consistent with the narrative review design, no formal quality appraisal of individual studies was performed.

Second, the selection of six sentinel findings was intentional to maintain clinical usability; however, other potentially relevant findings (e.g., supernumerary teeth, specific facial dysmorphisms, or skeletal asymmetries) were not included and may warrant incorporation in future iterations.

Third, the framework has not yet undergone prospective validation. Its diagnostic performance, including sensitivity, specificity, and impact on referral patterns, remains to be established in clinical settings. Consequently, use of the framework may result in false-positive triggers (i.e., referral of patients without an underlying syndrome) and potential over-referral to specialist services. Many sentinel findings demonstrate limited diagnostic specificity when considered in isolation, and phenotypic expression of syndromic conditions may vary considerably between individuals, increasing the risk of both false-positive referrals and missed presentations. These risks are inherent to screening-oriented approaches and should be balanced against the potential benefit of earlier recognition and timely referral. Furthermore, over-reliance on threshold-based reasoning could inadvertently reduce reliance on broader clinical judgment; therefore, the framework is intended as an adjunct to, rather than a replacement for, comprehensive clinical assessment. The diagnostic accuracy, inter-clinician reliability, sensitivity/specificity, and real-world clinical utility of the framework remain unknown and require prospective evaluation before routine clinical implementation. In addition, false-positive referrals may contribute to unnecessary patient anxiety and healthcare burden, which should be carefully balanced against the potential benefits of earlier recognition and appropriate specialist evaluation.

Fourth, the framework may perform less effectively in certain clinical scenarios. For example, assessment may be challenging in patients with incomplete dentition (e.g., young children, mixed dentition, edentulous adults), where findings such as hypodontia or macroglossia may be difficult to evaluate. Similarly, interpretation may be complicated in populations with a high baseline prevalence of isolated findings (e.g., physiological oral pigmentation or familial craniofacial traits). In such situations, clinicians should interpret findings within the broader clinical context.

Finally, genetic diversity, healthcare infrastructure, and referral pathways may influence the applicability of the framework across different populations and healthcare settings. Adaptation to local clinical contexts may therefore be necessary. Implementation in routine dental practice may also face practical challenges, including limited consultation time, variability in clinician familiarity with rare syndromes, and differences in access to multidisciplinary referral pathways across healthcare settings.

## 7 Comparison with existing frameworks

Existing literature on syndromic recognition in dentistry is predominantly syndrome-centric, describing oral and craniofacial manifestations associated with specific syndromes. In contrast, the present framework adopts a feature-centric approach, beginning with commonly encountered oral and craniofacial findings and providing a structured analytical pathway to support clinical suspicion and referral.

Existing approaches to syndromic recognition include clinical gestalt, syndrome-specific reference resources, and AI-assisted phenotyping tools (e.g., Face2Gene). However, these approaches are often dependent on specialist expertise or are not specifically designed for structured chairside use by general dental practitioners. In contrast, the proposed framework offers a low-technology, feature-based cognitive aid intended to support structured clinical reasoning during routine dental examination. To our knowledge, existing dental literature has not clearly operationalised sentinel thresholds, an explicit pattern-completion step, and a structured four-step reasoning pathway within a unified framework for chairside syndromic recognition.

The proposed framework offers several novel contributions. First, it identifies a focused set of sentinel oral and craniofacial findings with evidence-informed thresholds for suspicion. Second, it explicitly differentiates primary referral triggers (high-risk findings/red flags) from supportive findings used for pattern completion. Third, it integrates a four-step analytical reasoning pathway intended to reduce cognitive bias and support more structured clinical decision-making. Finally, it provides practical referral guidance aimed at facilitating earlier recognition and multidisciplinary evaluation when syndromic conditions are suspected.

Prospective validation studies are needed to determine the diagnostic accuracy, referral impact, and clinical utility of the framework in real-world dental settings.

## 8 Future directions

Prospective validation of the framework is essential before routine clinical implementation. Future research should follow a stepwise approach:

- **Content validation** using structured expert consensus methods (e.g., Delphi methodology) to refine sentinel findings, thresholds, and referral criteria.
- **Retrospective case–control studies** comparing confirmed syndromic and non-syndromic cases to estimate diagnostic performance (e.g., sensitivity, specificity, positive predictive value).
- **Inter-clinician reliability studies** (e.g., kappa statistics) to assess consistency of framework application among general dental practitioners.
- **Prospective pilot studies** in general dental settings to evaluate real-world clinical utility, referral appropriateness, feasibility, and potential effects on time to syndromic recognition.

Integration with digital decision-support systems may further enhance usability. Emerging technologies such as AI-based facial phenotyping tools (e.g., Face2Gene, GestaltMatcher) may complement pattern recognition, although their role in routine dental practice remains exploratory (17). The framework may also be expanded to include additional sentinel findings as evidence evolves. Its educational value could be evaluated through structured assessments such as objective structured clinical examinations or script concordance testing (54).

## 9 Conclusions

General dental practitioners are uniquely positioned to identify early oral manifestations of syndromic conditions. However, in the absence of a structured approach, these findings are often interpreted as normal variation. The framework presented in this review offers a practical, cognitively informed method to support clinical suspicion and decision-making under uncertainty. By integrating epidemiological context, evidence-informed thresholds, targeted evaluation, and clear referral pathways, it may enable dentists to play a more effective role in early syndromic recognition and interdisciplinary care.

## References

[1] Salerno C D'Avola V Oberti L Almonte E Bazzini EM Tartaglia GM. Rare genetic syndromes and oral anomalies: a review of the literature and case series with a new classification proposal. Children. (2021) 9(1):12. 10.3390/children9010012 35053637 PMC8774676

[2] Bartzela TN Carels C Maltha JC. Update on 13 syndromes affecting craniofacial and dental structures. Front Physiol. (2017) 8:1038. 10.3389/fphys.2017.01038 29311971 PMC5735950

[3] Țenț A Iurcov R Moca AE Moca RT Țig IA Matei RI. Oral manifestations in adolescents with genetic syndromes: a retrospective cross-sectional study. J Clin Med. (2025) 14(20):7217. 10.3390/jcm14207217 41156087 PMC12565033

[4] Torres-Avalos CG Cuevas-González JC Cuevas-González MV García-Calderón AG. Most frequent oral and craniofacial clinical characteristics associated with syndromes: a systematic review. Oral Surg Oral Med Oral Pathol Oral Radiol. (2025) 139(1):e29. 10.1016/j.oooo.2024.10.236

[5] Calvo F Karras BT Phillips R Kimball AM Wolf F. Diagnoses, syndromes, and diseases: a knowledge representation problem. AMIA Annu Symp Proc. (2003) 2003:802. 14728307 PMC1480257

[6] Croskerry P. A universal model of diagnostic reasoning. Acad Med. (2009) 84(8):1022–8. 10.1097/ACM.0b013e3181ace703 19638766

[7] Norman GR. The epistemology of clinical reasoning: perspectives from philosophy, psychology, and neuroscience. Acad Med. (2000) 75(10):S127–35. 10.1097/00001888-200010001-00041 11031197

[8] Bhattacharya SDS Ghosh R Mitra Ghosh D Roy P Pal R. Cognitive biases in dentistry: enhancing decision-making through psychological insights. J Contemp Clin Pract. (2025) 11(7):956–62.

[9] Murdoch AIK Blum J Chen J Baziotis-Kalfas D Dao A Bai K. Determinants of clinical decision making under uncertainty in dentistry: a scoping review. Diagnostics. (2023) 13(6):1076. 10.3390/diagnostics13061076 36980383 PMC10047498

[10] Torres-Avalos CGG-CAG Cuevas-Gonzalez JC Cuevas-Gonzalez MV Espinosa-Cristobal LF Barrio-Soule RA Hernandez-Cepeda OA. Most frequent oral and craniofacial clinical characteristics associated with syndromes. Int J Odontostomat. (2025) 19(4):369–75. 10.4067/s0718-381x2025000400369

[11] Buchanan EP Xue AS Hollier LH Jr. Craniofacial syndromes. Plast Reconstr Surg. (2014) 134(1):128e–53e. 10.1097/PRS.0000000000000308 25028828

[12] Luo E Liu H Zhao Q Shi B Chen Q. Dental-craniofacial manifestation and treatment of rare diseases. Int J Oral Sci. (2019) 11(1):9. 10.1038/s41368-018-0041-y 30783081 PMC6381182

[13] Deo JK. Craniofacial syndromes: literature review and a proposed classification. J Indira Gandhi Inst Med Sci. (2022) 8(2). 10.4103/jigims.jigims_9_22

[14] Cianci P Selicorni A. Gestalt diagnosis” for children with suspected genetic syndromes. Ital J Pediatr. (2015) 41(2):A16. 10.1186/1824-7288-41-S2-A16

[15] Suri M. Craniofacial syndromes. Semin Fetal Neonatal Med. (2005) 10(3):243–57. 10.1016/j.siny.2004.12.002 15927879

[16] Carrer A Romaniello MG Calderara ML Mariani M Biondi A Selicorni A. Application of the Face2Gene tool in an Italian dysmorphological pediatric clinic: retrospective validation and future perspectives. Am J Med Genet A. (2024) 194(3):e63459. 10.1002/ajmg.a.63459 37927205

[17] Hsieh TC Bar-Haim A Moosa S Ehmke N Gripp KW Pantel JT. Gestaltmatcher facilitates rare disease matching using facial phenotype descriptors. Nat Genet. (2022) 54(3):349–57. 10.1038/s41588-021-01010-x 35145301 PMC9272356

[18] Kováč P Jackuliak P Bražinová A Varga I Aláč M Smatana M. Artificial intelligence-driven facial image analysis for the early detection of rare diseases: legal, ethical, forensic, and cybersecurity considerations. AI. (2024) 5(3):990–1010. 10.3390/ai5030049

[19] Khalaf K Miskelly J Voge E Macfarlane TV. Prevalence of hypodontia and associated factors: a systematic review and meta-analysis. J Orthod. (2014) 41(4):299–316. 10.1179/1465313314Y.0000000116 25404667

[20] Rauniyar A Clarke J Law C Prabhu N. Review of hypodontia and its impact on oral health related quality of life in children. Pediatr Dent J. (2025) 35(3):100356. 10.1016/j.pdj.2025.100356

[21] Castilho NL Resende KKM Santos JAD Machado RA Coletta RD Guerra ENS. Oligodontia in the clinical Spectrum of syndromes: a systematic review. Dent J. (2023) 11(12):279. 10.3390/dj11120279 PMC10742796 38132417

[22] Dixit S Rokaya D Subra MNM. Prevalence and patterns of non-syndromic hypodontia in permanent dentition among the Nepalese population: a radiographic study and literature review. Open Dent J. (2025) 19(1). 10.2174/0118742106391120250430093302

[23] Reynolds L Dave M Tattar R Barry S. Inherited dental anomalies—part 1: enamel defects. Fac Dent J. (2024) 15(3):98–106. 10.1308/rcsfdj.2024.31

[24] Xu S Zhao C Jia L Ma Z Zhang X Shi H. Relationship between preterm, low birth weight, and development defects of enamel in the primary dentition: a meta-analysis. Front Pediatr. (2022) 10:975340. 10.3389/fped.2022.975340 36440332 PMC9684462

[25] Schwendicke F Elhennawy K Reda S Bekes K Manton DJ Krois J. Global burden of molar incisor hypomineralization. J Dent. (2018) 68:10–8. 10.1016/j.jdent.2017.12.002 29221956

[26] Boutiou E Ziogas IA Giannis D Doufexi A-E. Hereditary gingival fibromatosis in children: a systematic review of the literature. Clin Oral Investig. (2021) 25(6):3599–607. 10.1007/s00784-020-03682-x 33188467

[27] Carli E Lardani L Fitzgibbon R Fambrini E Bagattoni S. Periodontology part 3: hereditary gingival fibromatosis (HGF): from diagnosis to treatment in the paediatric age. Eur J Paediatr Dent. (2022) 23(3):249–50. 10.23804/ejpd.2022.23.03.13 36172902

[28] Doufexi A Mina M Ioannidou E. Gingival overgrowth in children: epidemiology, pathogenesis, and complications. A literature review. J Periodontol. (2005) 76(1):3–10. 10.1902/jop.2005.76.1.3 15830631

[29] Costa CRR Braz SV de Toledo IP Martelli-Júnior H Mazzeu JF Guerra ENS. Syndromes with gingival fibromatosis: a systematic review. Oral Dis. (2021) 27(4):881–93. 10.1111/odi.13369 32335995

[30] De Falco D Della Vella F Scivetti M Suriano C De Benedittis M Petruzzi M. Non-plaque induced diffuse gingival overgrowth: an overview. Appl Sci. (2022) 12(8):3731. 10.3390/app12083731

[31] Neville BW. Oral and Maxillofacial Pathology. 3rd ed. St Louis, Mo: Saunders/Elsevier (2009).

[32] Chapple ILC Mealey BL Van Dyke TE Bartold PM Dommisch H Eickholz P. Periodontal health and gingival diseases and conditions on an intact and a reduced periodontium: Consensus report of workgroup 1 of the 2017 World Workshop on the Classification of Periodontal and Peri-Implant Diseases and Conditions. (1943-3670 (Electronic). 10.1002/JPER.17-0719 29926944

[33] Ferreira LDS Calderipe CB Maass JB Carrard VC Martins MD Abreu LG. Oral pigmented lesions in syndromic individuals: a systematic review. Oral Dis. (2022) 28(3):531–40. 10.1111/odi.13769 33394507

[34] Pinna R Cocco F Campus G Conti G Milia E Sardella A. Genetic and developmental disorders of the oral mucosa: epidemiology; molecular mechanisms; diagnostic criteria; management. Periodontol 2000. (2019) 80(1):12–27. 10.1111/prd.12261 31090139

[35] Rotbeh A Kazeminia M Kalantari M Rajati F. Global prevalence of oral pigmentation and its related factors: a systematic review and meta-analysis. J Stomatol Oral Maxillofac Surg. (2022) 123(5):e411–e24. 10.1016/j.jormas.2022.01.009 35066171

[36] Sreeja C Ramakrishnan K Vijayalakshmi D Devi M Aesha I Vijayabanu B. Oral pigmentation: a review. J Pharm Bioallied Sci. (2015) 7(2):S403–8. 10.4103/0975-7406.163471 26538887 PMC4606629

[37] Topouzelis N Iliopoulos C Kolokitha OE. Macroglossia. Int Dent J. (2011) 61(2):63–9. 10.1111/j.1875-595X.2011.00015.x 21554274 PMC9374813

[38] Shlobin NA Baticulon RE Ortega CA Du L Bonfield CM Wray A. Global epidemiology of craniosynostosis: a systematic review and meta-analysis. World Neurosurg. (2022) 164:413–23.e3. 10.1016/j.wneu.2022.05.093 35636659

[39] Katouni K Nikolaou A Mariolis T Protogerou V Chrysikos D Theofilopoulou S. Syndromic craniosynostosis: a comprehensive review. Cureus. (2023) 15(12):e50448. 10.7759/cureus.50448 38222144 PMC10785997

[40] Stanbouly D Lee KC Chuang S-K. A narrative review of craniofacial deformities. Front Oral Maxillofac Med. (2024) 6:2022. 10.21037/fomm-21-85

[41] Sawh-Martinez R Steinbacher DM. Syndromic craniosynostosis. Clin Plast Surg. (2019) 46(2):141–55. 10.1016/j.cps.2018.11.009 30851747

[42] Faria-Teixeira MC Tordera C Salvado e Silva F Vaz-Carneiro A Iglesias-Linares A. Craniofacial syndromes and class III phenotype: common genotype fingerprints? A scoping review and meta-analysis. Pediatr Res. (2024) 95(6):1455–75. 10.1038/s41390-023-02907-5 38347173 PMC11126392

[43] Padula MA Naing K Wenger TL Ahmad I Coghill CH 3rd Wild KT. Spectrum of disease in hospitalized newborns with congenital micrognathia: a cohort of 3,236 infants at North American tertiary-care intensive care units. J Pediatr. (2024) 265:113799. 10.1016/j.jpeds.2023.113799 37879601 PMC10872910

[44] Singh DJ Bartlett SP. Congenital mandibular hypoplasia: analysis and classification. (1049-2275 (Print)). 10.1097/00001665-200503000-00017 15750428

[45] Zimmerer RM Sander AK Schönfeld A Lethaus B Gellrich N-C Neuhaus M-T. Congenital mandibular hypoplasia: patient-specific total joint replacement as a line extension in the treatment of complex craniofacial anomalies. J Maxillofac Oral Surg. (2023) 22(2):410–8. 10.1007/s12663-022-01780-9 37122781 PMC10130262

[46] Amberger JS Bocchini CA Schiettecatte F Scott AF Hamosh A. OMIM.Org: online Mendelian inheritance in man (OMIM®), an online catalog of human genes and genetic disorders. Nucleic Acids Res. (2015) 43(Database issue):D789–98. 10.1093/nar/gku1205 25428349 PMC4383985

[47] Ardila CM Álvarez-Martínez E. Dental and maxillofacial manifestations of axenfeld-rieger syndrome: presentation of a case in a 5-year-old girl. Case Rep Dent. (2022) 2022:4348264. 10.1155/2022/4348264 35957627 PMC9363210

[48] Arora V Agrawal KK Mishra A Chandra A. Witkop’s syndrome: a case report. J Oral Biol Craniofac Res. (2016) 6(1):79–81. 10.1016/j.jobcr.2015.07.003 26937375 PMC4756068

[49] Glick M Greenberg MS Lockhart PB Challacombe SJ. Burket’s Oral Medicine. Hoboken, NJ: Wiley (2021).

[50] Dehailan A Martinez-Mier L A E. Evidence on the association of overall dietary factors, selected environmental, medical, demographic, and biological factors and developmental defects of enamel, including MIH and enamel fluorosis. Front Oral Health. (2025) 6:1616109. 10.3389/froh.2025.1616109 41458464 PMC12738894

[51] Sivapathasundharam B. Shafer’s Textbook of Oral Pathology- E-Book. Gurgaon: Elsevier India (2024).

[52] Ciurea AV Toader C. Genetics of craniosynostosis: review of the literature. J Med Life. (2009) 2(1):5–17. 20108486 PMC5051481

[53] Paradowska-Stolarz AM Ziomek M Sluzalec-Wieckiewicz K Duś-Ilnicka I. Most common congenital syndromes with facial asymmetry: a narrative review. Dent Med Probl. (2024) 61(6):925–32. 10.17219/dmp/186086 39496100

[54] Custers EJ. Thirty years of illness scripts: theoretical origins and practical applications. Med Teach. (2015) 37(5):457–62. 10.3109/0142159X.2014.956052 25180878

[55] Feller L Lemmer J Nemutandani MS Ballyram R Khammissa RAG. Judgment and decision-making in clinical dentistry. J Int Med Res. (2020) 48(11):300060520972877. 10.1177/0300060520972877 33249958 PMC7708710

